# Characteristics of *Staphylococcus aureus* Bacteraemia and Predictors of Early and Late Mortality

**DOI:** 10.1371/journal.pone.0170236

**Published:** 2017-02-02

**Authors:** Matteo Bassetti, Maddalena Peghin, Enrico Maria Trecarichi, Alessia Carnelutti, Elda Righi, Paola Del Giacomo, Filippo Ansaldi, Cecilia Trucchi, Cristiano Alicino, Roberto Cauda, Assunta Sartor, Teresa Spanu, Claudio Scarparo, Mario Tumbarello

**Affiliations:** 1 Infectious Diseases Division, Santa Maria Misericordia University Hospital, Udine, Italy; 2 Institute of Infectious Diseases, Catholic University of the Sacred Heart, Rome, Italy; 3 IRCCS AOU San Martino IST, Department of Health Sciences, University of Genoa; Genoa, Italy; 4 Microbiology Unit, Santa Maria Misericordia University Hospital, Udine, Italy; 5 Institute of Microbiology, Catholic University of the Sacred Heart, Rome, Italy; Universitatsklinikum Munster, GERMANY

## Abstract

We aimed to describe the characteristics of patients with *Staphylococcus aureus* bacteremia and to evaluate the risk factors associated with early (7-day) and late (30-day) mortality. We performed an observational study including all consecutive episodes of *Staphylococcus aureus* bacteremia diagnosed at two Italian university hospitals during 2010–2014. A total of 337 patients were included. Mean age was 69 years (range, 57–78) and 65% were males. Methicillin-resistant *S*. *aureus* (MRSA) was identified in 132/337 (39%)cases. Overall 7- and 30-day mortality were 13% and 26%, respectively. Early mortality was associated with increased Charlson scores (OR 1.3, 95% CI 1.1–1.5), MRSA bacteremia (OR 3.2, 95% CI 1.4–8.1), presentation with septic shock (OR 13.5, 95% CI 5.4–36.4), and occurrence of endocarditis (OR 4.5, 95%CI 1.4–14.6). Similar risk factors were identified for late mortality, including increased Charlson scores (OR 1.2, 95% CI 1.1–1.4), MRSA bacteremia (OR 2.1, 95% CI 1.2–3.9), presentation with septic shock (OR 4, 95%CI 1.7–9.7), occurrence of endocarditis (OR 3.8, 95% CI 1.4–10.2) as well as Child C cirrhosis (OR 3.9, 95% CI 1.1–14.4) and primary bacteremia (OR 2.5, 95%CI 1.3–5). Infectious disease consultation resulted in better outcomes both at 7 (OR 0.1, 95% CI 0.05–0.4) and at 30 days (OR 0.4, 95% CI 0.2–0.7). In conclusion, our study highlighted high rates of MRSA infection in nosocomial *Staphylococcus aureus* bacteremia. Multiple comorbidities, disease severity and methicillin-resistance are key factors for early and late mortality in this group. In patients with *Staphylococcus aureus* bacteremia, infectious disease consultation remains a valuable tool to improve clinical outcome.

## Introduction

The distribution of methicillin-sensitive (MSSA) and methicillin-resistant *S*. *aureus* (MRSA) bacteremia varies substantially by geographical region [1] [2] [3] [4] [5]. *Staphylococcus aureus*, however, remains a leading cause of community- and healthcare-associated bacteremia worldwide [3] [5]. Despite efforts to reduce its incidence, *S*. *aureus* bacteremia (SAB) remains frequent and associated with mortality rates up to 25% [3]. SAB acquisition and outcome are affected by patients’ comorbidities, infection site, and methicillin-resistance [6] [7]. In this patient population, evidence-based bundle interventions demonstrated to have a positive impact in clinical management and outcome [8]. In particular, results from a recent metanalysis suggested that infectious diseases (ID) consultation might improve clinical care and impact hospital survival in patients with SAB [8]. Most of the studies, however, focused on predictors of 30- and 60 day-mortality even if high early (≤ 7 day) mortality rates have been reported among patients with SAB [6] [7] [8] [9] [10, 11] [12].Aim of the study was to describe the epidemiology and clinical characteristics of patients with SAB and assess the prognostic factors for early (7-day) and late (30-day) mortality.

## Material and Methods

### Study design, setting and patients

A retrospective, observational, cohort study was performed in two tertiary care university hospitals in Italy that admit approximately 90,000 patients per year. All episodes of clinically significant SAB in adult patients (aged 18 years and older) between January 2011 and December 2014 were included. Patients were followed in the participant centers until discharge or death. Cases were detected through electronic databases, using a standardized protocol including age, sex, comorbidities, source of infection, setting of acquisition, antimicrobial susceptibility, antibiotic treatment, and clinical outcome. This study was approved by the local institutional review board (Comitato Etico Regionale Unico). Because of the retrospective nature of the study, the requirement for informed consent was waived.

### Definitions

SAB was defined as the presence of ≥1 positive blood culture for *S*. *aureus* in patients with signs and symptoms consistent with an infection [7]. Onset of SAB was defined as the date of collection of the first blood culture yielding positivity for *S*.*aureus*. Only the first clinically significant episode of infection with SAB for each patient was included in the analysis.

SAB was defined as hospital-acquired (HA) if the first positive blood culture was performed more than 48 hours after admission either to the intensive care unit (ICU) or to another hospital ward [13]. Other SAB were classified as HCA or CA according to previously reported definitions [14].

In the presence of a laboratory-confirmed bloodstream infection, bacteremia of unknown source or primary bacteremia was defined when its origin could not been established after careful examination of clinical signs, microbiological findings, and imaging results. Otherwise, 72 hours from blood culture collection or by the presence of an ID physician responsible for the patient’s management. At both hospitals, ID service consultation was available and optional.

Empirical therapy was defined as adequate when at least one active antimicrobial agent was administered at recommended doses according to susceptibility data within the first 48 hours after the blood cultures were performed. Antibiotic de-escalation was defined as switch to a narrower spectrum agent [15]. Targeted antibiotic therapy was considered adequate when at least one active antimicrobial agent was administered, according to susceptibility data, at recommended doses after 96 hours following blood cultures collection. Regimens were classified as monotherapy or combination therapy depending on the number of active drugs included. Overall mortality was determined at 7 days (early mortality) and at 30 days (late mortality) after the first blood culture.

### Blood cultures and microbiology analysis

Identification of isolates and antimicrobial susceptibility profiles were obtained through VITEK 2 (bioMérieux Inc., Hazelwood, MO, USA). The Aris Sensititre instrument for incubating and reading susceptibility plates (Trek Diagnostic Systems Inc., Independence, OH) was used according to European Committee on Antimicrobial Susceptibility Testing (EUCAST) breakpoints, and Etest strips (BioMerieux) were used to confirm antibiotic resistance [16]

### Statistical analysis

Continuous and categorical data were reported as median, 25th and 75th percentile and frequency distributions, respectively. The Wilcoxon test was used to determine if differences existed between groups for continuous variables, respectively. Categorical variables were evaluated using chi-square or, when appropriate, the two-tailed Fisher’s exact test. Multiple logistic regression analysis was performed to identify risk factors that were associated with 7- and 30-day hospital mortality [JMP, SAS, NC, USA). Covariates that were significant at 0.10 in the univariate analysis and therapy-related variables were further evaluated for inclusion in multivariable regression models, using a backward stepwise algorithm. Given the high number of potential independent variables, a backward stepwise algorithm was used to identify the best-fitting subset of variables for use in the final multivariable regression model. In particular, Akaike’s Information Criterion (AIC) was used to assess the models’ fit and the model with the lowest AIC was selected for the multivariable analysis. All tests were two-tailed, and a P value less than 0.05 was determined to represent statistical significance.

## Results and Discussion

### Population characteristics

During the four-year study period, 337 episodes of SAB were diagnosed (see the database in S1 and S2 Data). The demographic characteristics and underlying conditions of these patients are summarised in Table 1.

**Table 1:** Characteristics of patients with staphylococcal bacteremia caused by MRSA compared with patients with staphylococcal bacteremia caused by MSSA.

	MRSAn = 132(39.2%)	MSSAn = 205 (60.9%)	P-value
**Characteristic**			
**Demographic data**			
Age, years (median, IQR)	69 (56–77)	68 (58–79)	0.88
Gender, male	92/132 (69.7)	127/205 (62)	0.15
BMI (kg/m^2^) (median, IQR)	24.6 (22.2–28.7)	24.5 (22.5–27.6)	0.76
**Baseline disease**			
AAC score (median, IQR)	6 (2–6)	5 (2–7)	0.52
Solid organ recipient	3/132 (2.3)	10/205 (4.9)	0.23
Solid cancer	34/132 (25.8)	45/205 (22)	0.42
Hematologic malignancies	3/132 (2.3)	21/205 (10.2)	0.006
Neutropenia (<500 PMN/mm3)	9/132 (6.8)	10/205 (4.9)	0.45
*Dialysis*	17/132 (12.9)	21/205 (10.2)	0.46
Cr clearance (ml/min) (median, IQR)	90.4 (39.2–133.1)	92.8 (52.2–136.6)	0.88
Diabetes	42/132 (31.8)	73/205 (35.6)	0.47
Cirrhosis	18/132(13.6)	35/205(17.1)	0.40
Child score [32]			
A	1/6 (16.7)	5/19 (26.3)	0.64
B	1/6 (16.7)	3/19 (15.8)	0.96
C	4/6 (66.6)	11/19 (57.9)	0.71
**Baseline treatment**			
Immunosuppressive therapy	11/132 (8.3)	12/205 (5.9)	0.38
Steroids therapy	18/132 (13.6)	32/205 (15.6)	0.62
Cancer chemotherapy (<30 days)	10/132 (7.6)	21/205 (10.2)	0.41
**Other risk factors**			
Active consumption of drugs intravenously	9/132 (6.8)	10/205 84.9)	0.45
CVC (>72h)	70/132 (53)	73/205 (35.6)	0.002
Other intravascular device (>72h)	10/132 (7.6)	5/205 (2.4)	0.03
Abdominal drainage catheter	15/132 (11.4)	9/205 (4.4)	0.02
Urinary catheter (>72h)	54/132 (40.9)	64/205 (31.2)	0.07
Recent antimicrobial therapy (<30 days)	60/132 (45.5)	63/205 (30.7)	0.006
Surgery (<30days)	34/132 (25.8)	30/205 (14.6)	0.01
Prior ICU care (<90 days)	17/132 (12.9)	14/205 (6.8)	0.06
**SAB data**			
**Setting of acquisition**			
Community acquired	2/132 (1.5)	29/205 (14.2)	<0.001
Health care associated	40/132 (30.3)	53/205 (25.8)	0.37
Hospital acquired	90/132 (68.2)	123/205 (60)	0.13
**Source of infection**			
Unknown	38/132 (28.8)	106/205 (51.7)	<0.001
CVC	23/132 (17.4)	16/205 (7.8)	0.007
Pulmonary	13/132 (9.9)	7/205 (3.4)	0.02
Endocarditis	23/132(17.4)	14/205(6.8)	0.002
Skin soft tissues	15/132 (11.4)	27/205 (13.2)	0.61
Bone	1/132 (0.8)	7/205 (3.4)	0.12
Surgical site	8/132 (6.1)	10/205 (4.9)	0.65
Urinary	5/132 (3.8)	8/205 (3.9)	0.95
Other	6/132 (4.5)	10/205 (4.9)	0.88
**Clinical presentation**			
Septic shock	25(18.9%)	20(9.8)	0.03
ICU admission <72h	10/132 (7.6)	11/205 (5.4)	0.41
**Therapeutic management**			
Infectious disease consultation	57/132 (43.2)	117/204 (57.4)	0.01
CVC removal in patients with CVC >72h	54/70 (77.1)	57/73 (78.1)	0.89
Source control in patients with known source of infection	34/94 (36.2)	40/99 (40.4)	0.54
Empiric or targeted antimicrobial therapy	126/132 (95.5)	205/205 (100)	0.002
Empiric antimicrobial therapy	116/132 (87.9)	190/205 (92.7)	0.14
Empiric Daptomycin	7/132 (5.3)	25/205 (12.2)	0.04
Empiric Glycopeptide	25/132 (18.9)	25/205 (12.2)	0.09
Targeted antimicrobial therapy	124/132 (93.9)	197/205 (96.1)	0.36
De-escalation	35/130 (26.9)	75/203 (37)	0.06
Start of therapy/de-escalation (days) (median, IQR)	5 (3–10)	5 (3–7)	0.84
Adequate initial (< 48 h) therapy	32/132 (24.2)	189/205 (92.2)	<0.001
If adequate initial therapy, combination therapy	10/32 (31.3)	85/189 (45)	0.15
Adequate targeted (> 96 h) therapy	120/132 (90.9)	204/205 (99.5)	<0.001
Duration of therapy (days) (median, IQR)	16 (8–22)	17 (14–29)	0.008
Duration of hospitalization (days) (median, IQR)	32 (20–53)	28 (14.5–47.5)	0.18

Data are no. of positive results / total number studied (%), unless otherwise indicated.

AAC, age adjusted Charlson; BMI, body mass index; Cr, creatinine; CVC, central venous catheter; ICU, intensive care unit; IQR, interquartile range; y, years; MRSA, methicillin resistant S*taphylococcus aureus*; SAB, *Staphylococcus aureus* bacteremia.

Of all cases of SAB, 9.2% were CA, 27.6% HCA and 63.2% were nosocomial. Among nosocomial infections, the majority was acquired in a medical ward (63.9%). Most common sites of infection were skin and soft tissue (12.5%), CVC-related infections (11.6%), endocarditis (10.9%), respiratory tract infections (5.9%), surgical site (5.3%), urinary tract (3.9%) and bone infections (2.4%). In 144 (42.7%) patients a clear source of infection was not identified.

### Antimicrobial resistance and therapeutic management

MRSA isolates were identified in 132/337 cases (39.2%), mostly among HCA (43%, 40/93) and HA (42.3%, 90/213) (Table 1). MRSA isolates were more common among patients with indwelling devices (e.g., CVC and abdominal drainage catheter), recent surgery, and antimicrobial therapy compared to MSSA (Table 1). Patients with MRSA bacteremia had higher severity at presentation (e.g., septic shock) and greater proportion of pulmonary and CVC-related infections compared to MSSA bacteremia. Adequate empiric and definitive antibiotic therapy, as well as use of empiric daptomycin, were lower in patients with MRSA isolates compared to MSSA isolates. The comparison between patients with MRSA and MSSA SAB is reported in Table 1.

An ID specialist was consulted in 51.6% of cases within 72 hours of the onset of bacteremia. Once the diagnosis of SAB was suspected, 90.8% (306/337) of patients received empiric systemic antibiotic therapy. An empiric combination therapy was prescribed in 37.9% (116/306). A beta-lactam represented the most used empiric regimen (189/306, 61.8%), followed by glycopeptides (53/306, 17.3%) and fluoroquinolones (40/306, 13.1%). Empirical therapy was considered adequate in 219 (64.9%) episodes, and in 43.3% of cases included a combination treatment. Overall, definitive antibiotic therapy was appropriate in 96.4% (325/337).

### Outcome

Twenty-one patients (6.2%) were admitted to the ICUs ≤ 72 hours after the first positive blood culture. Seven- and 30-day mortality were 13.1% (44 out of 337) and 25.5% (86 out of 337), respectively, and were significantly higher for patients with MRSA infections compared to MSSA infections at both time-periods (Table 2). Compared to survivors, patients with SAB who died within 7 or 30 days after first positive blood culture were more likely to be older, have a higher age-adjusted Charlson score, have a pulmonary or cardiac source of infection, present with septic shock, and be admitted to the ICU within 72 hours from SAB diagnosis. In contrast, mortality was lower in patients with a urinary tract source of infection and undergoing catheter removal. Lower mortality rates were also reported in patients receiving adequate therapy, patients treated with daptomycin or undergoing de-escalation therapy, and those evaluated by an ID specialist. Risk factors for mortality at univariate analysis of are shown in Table 2.

**Table 2:** Univariate analysis of risk factors for mortality at 7 and 30 days in patients with *Staphylococcus aureus* bacteremia.

	7-day			30-day		
Characteristic	Survivors n = 293 (86.9%)	Non Survivors n = 44 (13.1%)	P-value	Survivors n = 251 (74.5%)	Non Survivors n = 86 (25.5%)	P-value
Age, years (median, IQR)	68 (57–77)	75 (60–79)	0.03	68 (56–76.3)	74 (59–82)	0.003
Gender, male	193 (65.8)	26 (59.1)	0.36	164 (65.3)	55 (64)	0.8
BMI (kg/m^2^) (median, IQR)	24.6 (22.5–27.8)	24.6 (21.9–27.4)	0.7	24.6 (22.5–28.1)	24.6 (21.7–26.3)	0.26
AAC score (median, IQR)	5 (2–6)	6 (4–8)	0.01	5 (2–6)	6 (4–8)	<0.001
Solid organ recipient	12 (4.1)	1 (2.3)	1.00	13 (5.1)	1(1.2)	0.2
Solid cancer	64 (21.8)	14(31.8)	0.15	56 (22.3)	22 (25.6)	0.54
Hematologic malignancies	19 (6.5)	5(11.4)	0.24	16 (6.3)	8 (9.3)	0.39
Neutropenia (<500 neutrophil count/mm3)	12 (4.1)	7 (15.9)	0.002	13 (5.1)	7 (8.1)	0.26
Dialysis	31 (10.6)	6(13.6)	0.55	29 (11.5)	9 (10.5)	0.82
Cr clearance (ml/min) (median, IQR)	93.3 (51.8–136)	61.4 (29.5–109.9)	0.21	97 (57–138)	63 (29–105)	0.03
Diabetes	104 (35.5)	10 (22.7)	0.09	81 (32.2)	33 (38.4)	0.29
Cirrhosis	49 (16.7)	7(15.9)	0.89	39 (15.5)	15 (17.4)	0.66
**Child score**			1.			
A	11/49 (22.5)	4/7 (57.1)	0.4	13 (33.3)	1 (6.7	0.3
B	8/49 (16.3)	0	1.0	10 (25.6)	0 (0)	0.12
C	30/49 (61.2)	3/7 (42.9)	1.0	16 (41.2)	14 (93.3)	0.02
Immunosuppressive therapy	21 (7.2)	2 (4.6)	0.75	21 (8.3)	2 (2.3)	0.05
Steroid therapy	43 (14.7)	7 (15.9)	0.84	36(14.3)	13 (15.1)	0.91
Cancer chemotherapy (<30 days)	27 (9.2)	4 (9.1)	1.00	25 (9.9)	7 (8.1)	0.66
Active consumption of drugs intravenously	14 (4.8)	5 (11.4)	0.08	13 (5.1)	6/86 (7)	0.56
CVC (>72h)	124 (42.3)	19 (43.2)	0.91	104 (41.4)	39 (45.4)	0.53
Other intravascular device (>72h)	13 (4.4)	2 (4.6)	1.00	14 (5.5)	1 (1.2)	0.13
Abdominal drainage catheter	18 (6.2)	6 (13.6)	0.07	18 (7.1)	6 (7)	0.92
Urinary catheter (>72h)	98 (33.4)	20 (45.5)	0.12	86 (34.2)	33 (38.4)	0.52
Recent antimicrobial therapy (<30 days)	101 (34.4)	21 (47.7)	0.09	83 (33.1)	39/86 (45.4)	0.05
Surgery (<30days)	53 (18.0)	10 (22.7)	0.47	51(20.3)	12/86 (14)	0.17
Prior ICU care (<90 days)	28 (9.6)	3 (6.8)	0.78	22 (8.7)	9/86 (10.5)	0.68
**Setting of acquisition**						
Community acquired	25 (8.5)	6 (13.6)	0.30	22 (8.7)	9(10.5	0.6
Health care associate	82 (28.0	11 (25)	0.6	68 (27.1)	24 (27.9)	0.96
Hospital acquired	185(63.5)	27 (61.4)	0.80	156(62.1)	53(61.6)	0.77
**Source of infection**						
Unknown	126 (43)	18 (40.9)	0.79	99 (39.4	43 (50)	0.09
CVC	35 (11.9)	4 (9.1)	0.58	31 (12.3)	8 (9.3)	0.55
Pulmonary	14 (4.8)	6 (13.6)	0.02	9 (3.5)	9 (10.5)	0.02
Endocarditis	24 (8.2	13 (29.6)	<0.001	22 (8.7)	15(17.4	0.03
Skin soft tissues	40 (13.7)	2 (4.6)	0.1	33 (13.1	9 (10.5	0.4
Bon	8 (2.7)	0 (0)	0.60	8 (3.1)	0 (0)	0.12
Surgical site	18 (6.1)	0 (0)	0.15	17 (6.8)	1 (12)	0.05
Urinary	13 (4.4	0 (0	0.23	13 (5.1)	0 (0	0.02
Other	15 (5.1)	1 (2.3)	0.70	15 (5.9)	1 (1.2)	0.08
MRSA	101(34.4)	30 (68.2)	<0.001	83 (33.1)	47 (54.7)	<0.001
Septic shock	20 (6.8)	23 (52.3)	<0.001	19(7.5)	24(27.9)	<0.001
ICU admission <72h	14(4.8)	7 (15.9)	0.005	10(3.9)	11 (12.8)	0.004
Infectious disease consultation	166 (56.7)	8 (18.2)	<0.001	147 (58.6)	27 (31.4)	<0.001
CVC removal in patients with CVC >72h	102/124 (82.2)	9/19 (47.3)	0.002	89/104 (85.6)	24/39(61.5)	0.002
Source control	68/167 (40.7)	6/26 (23.1)	0.09	60/152 (39.5)	14/43 (32.6)	0.41
Empiric antimicrobial therapy	267 (91.1)	39 (88.6)	0.54	228 (90.8)	78 (90.7)	0.92
Empiric Daptomycin	31 (10.6)	1 (2.3)	0.08	29 (11.5)	3 (3.5)	0.02
Empiric Glycopeptide	45 (15.3)	5 (11.3)	0.48	34 (13.5)	16 (18.6)	0.29
Targeted antimicrobial therapy	290 (98.9)	31 (70.5)	<0.001	248 (98.8)	74 (86.1)	<0.001
De-escalation therapy	105(35.8)	5 (11.6)	0.001	81 (32.2)	27 (31.4)	0.09
Start of therapy/de-escalation (days) (median, IQR)	5 (3–9)	2 (1.5–5.5)	0.07	5 (3–8)	4.5 (3–8)	0.58
Adequate therapy (< 48h)	201(68.6)	20 (45.5)	0.002	175 (69.7)	46 (53.5)	0.005
If adequate initial therapy, combination therapy	90/201 (44.8)	5/20 (25)	0.09	79/173 (45.7)	15/46 (32.6)	0.11
Adequate targeted therapy (> 96h)	288(98.2)	36 (81.8)	<0.001	247 (98.4)	79 (90.7)	0.001

Multivariate analysis identified as risk factors independently associated to both early and late fatal outcome a high age-adjusted Charlson score, the occurrence of endocarditis, septic shock at clinical presentation, and infections caused by MRSA. Other risk factors, only for 30-day mortality, included Child C cirrhosis and primary bacteremia. Consultation with an ID specialist and de-escalation therapy represented factors associated with a decreased risk of mortality at both timepoint and at 7-days, respectively (Table 3). ID consultation was associated to higher rates of adequate initial antibiotic regimen (71.8% vs. 57.6%, P = 0.006) and more frequent recommendation for the use of daptomycin in empiric therapy (53.2% vs. 30.5%, P = 0.001) (Table 4).

**Table 3:** Multivariate analysis of risk factors for mortality due to *Staphylococcus aureus* bacteremia at 7 and 30 days.

Characteristic	7-day mortality		30-day mortality	
	OR (95% CI)	P-value	OR (95% CI)	P-value
AAC score (median, IQR)	1.3 (1.1–1.5)	0.001	1.2 (1.1–1.4)	<0.001
Child score C	NS	NS	3.9 (1.1–14.4)	0.04
**Source of infection**				
Unknown	NS	NS	2.5 (1.3–5)	0.007
Endocarditis	4.5 (1.4–14.6)	0.01	3.8 (1.4–10.2)	0.009
MRSA	3.2 (1.4–8.1)	0.007	2.1 (1.2–3.9)	0.02
Septic shock	13.5 (5.4–36.4)	<0.001	4 (1.7–9.7)	0.01
Infectious disease consultation	0.1 (0.05–0.4)	<0.001	0.4 (0.2–0.7)	0.002
De-escalation therapy	0.3 (0.1–0.7)	0.01	NS	NS

AAC, age-adjusted Charlson score; IQR, interquartile range; MRSA, methicillin resistant S*taphylococcus aureus*.

OR, Odds ratio; NS = non significant.

**Table 4:** Univariate analysis of SAB with infectious disease consultation VS. SAB without ID consultation.

Variables	ID consultation n = 174 (51.6%)	Non-ID consultation n = 163 (48.4%)	p-value
**Adequate initial (< 48 h) therapy**	125 (71.8%)	94/163 (57.6%)	0.006
**Empiric therapy with Daptomycin**	31 (17.8%)	4/163 (2.5%)	<0.001
**Adequate initial (< 48 h) combination therapy**	66/124 (53.2%)	29/95 (30.5%)	0.001

Abbreviations: ID, infectious disease.

## Discussion

The proportion of MRSA has declined in the United States and in some European countries, including Italy, over the past years, but still remains a concern for public health and a major cause of nosocomial infections [1] [2]. In our study, we observed methicillin-resistance rates of about 40%, slightly higher than 34% reported for Italy by the European Antimicrobial Resistance Surveillance Network in 2014 [1]. In our study, MRSA accounted for most nosocomial and healthcare-associated infections but only for a limited proportion of community-acquired infections, stressing the importance of the clinical setting in defining patient populations at risk for MRSA bacteremia [17] [18]. Previous studies showed higher mortality rates among patients with MRSA bacteremia compared to MSSA, but the results are conflicting [7] [12] [19] [20] [21]. In our study, methicillin resistance was an independent risk factor for both early and late mortality survival. One reason could be represented by higher rates of inadequate treatment associated with MRSA compared to MSSA or by other potential confounding factors (e.g., disease severity, risk factors) associated with MRSA infections [10] [20] [22] [23]. Our study, in particular, showed lower rates of appropriate empiric and definitive therapy in patients with MRSA compared to MSSA infections.

We reported a significant survival benefit in SAB when ID service was involved. Specifically, ID consultation impacted long term as well as short-term mortality. ID consultation was also associated with an adequate initial therapy and empiric use of daptomycin. Our data support the growing body of evidence that ID consultation improves patients’ outcomes [8] [9] [24]. ID consultation can be optional or mandatory, depending on hospital policies, and its rate can vary from 33 to 88% [24] [25]. In our cohort, an ID specialist was consulted within 72 hours of the onset of bacteremia in only half of cases. The need to increase ID stewardship as part of hospital bundle interventions to positively impact patient care decisions and reduce SAB mortality should be carefully considered by all hospitals [8] [9] [11].

As previously reported, SAB mortality in our cohort increased with older age and presence of multiple comorbidities, including Child C cirrhosis [6] [7] [26] [27] [28] [29]. Severity at presentation also influenced SAB prognosis, being septic shock an independent risk factor for both early and late mortality [23].

We confirmed the occurrence of endocarditis as an independent risk factor for poor outcomes, including early mortality [6] [10]. Moreover, a relationship between death and the absence of an identified focus emerged for late mortality. In these instances, a better diagnostic definition of the source of infection could help to reach a better source control and improve patients’ management [30]

De-escalation therapy was also associated with reduced 7-days mortality. A reason could be represented by overall better clinical evolution or absence of multidrug resistance in patients undergoing de-escalation therapy [15].

Interestingly, combination therapy (mainly association of beta-lactams and glycopeptides) was often prescribed in our study (40% of all SAB) but did not impact the mortality. A reason could be that combination therapy was preferred to widen the antibacterial spectrum or to increase the synergistic bactericidal activity of the treatment in severe infections, this representing a confounding factor. The role of combined treatment in the setting of MSSA and MRSA bacteremia, however, appears promising but is yet to be defined [31]

Considerable early and late mortality rates were reported in our study (13% and 26%, respectively). Previous studies that analysed risk factors associated with SAB early mortality (e.g. within 2 to 9 days from the onset of the infection) showed mortality rates around 35% [10, 11] [12] [26]. Hence, early mortality represents a significant timepoint to analyse in SAB.

Our study presents several limitations. First, its retrospective, observational nature can limit the considerations on patient treatments. Second, we considered overall mortality rather than SAB-related mortality. We tried to overcome this bias using two timepoints for mortality, assuming that early mortality could have been more likely associated with SAB. Third, while not specific criteria for requesting an ID consultation have been established and followed, this option could be more likely requested in patients with more severe infections. To minimize the selection bias, we adjusted the data for confounders using multivariate analysis. Furthermore, molecular testing of *S*.*aureus* was not performed, but because of the retrospective nature of our study, this information was not available.

## Conclusions

In conclusion, *S*.*aureus* bacteremia represents a serious infection, associated with significant both early and late mortality. Methicillin resistance is associated mostly to nosocomial and health-care associated infections and may be a risk factor for mortality in patients with SAB. Clinicians should be aware of the severity of patients with *S*. *aureus* bacteremia, and infectious disease consultation should be always considered to improve patients’ outcomes.

## Supporting Information

S1 Data Anonymised database of Udine Hospital. (XLS)

S2 Data Anonymised database of Roma Hospital. (XLS)
